# Comparison Between Tracheal Wash and Bronchoalveolar Lavage Cytology for the Assessment of Exercise-Induced Pulmonary Hemorrhage (EIPH) in Racehorses

**DOI:** 10.3390/ani14223243

**Published:** 2024-11-12

**Authors:** Chiara Bozzola, Giulia Sala, Lorenzo Schinardi, Giovanni Stancari, Luca Stucchi, Francesco Ferrucci, Enrica Zucca

**Affiliations:** 1 Department of Veterinary Medicine and Animal Science, Università degli Studi di Milano, 26900 Lodi, Italy; chiara.bozzola@unimi.it (C.B.); giovanni.stancari@unimi.it (G.S.); francesco.ferrucci@unimi.it (F.F.); 2 Department of Veterinary Sciences, Università di Pisa, San Piero a Grado, 56122 Pisa, Italy; giulia.sala@unipi.it; 3 Veterinary Practitioner, 26121 Piacenza, Italy; lory.schin@gmail.com; 4 Department of Veterinary Medicine, Università degli Studi di Sassari, 07100 Sassari, Italy; lstucchi@uniss.it

**Keywords:** cytology, EIPH, horse, pulmonary bleeding, racehorses, standardbred, thoroughbred, Total Hemosiderin Score

## Abstract

Exercise-Induced Pulmonary Hemorrhage (EIPH) is a common pulmonary disease among racehorses, diagnosed by the detection of blood in the trachea after strenuous exercise or the presence of hemosiderophages in the bronchoalveolar lavage fluid (BALF). Although the latter is considered the most sensitive method to diagnose EIPH, it is perceived as a less practical and more invasive procedure compared to tracheal wash (TW) collection among racehorse trainers. The present retrospective study aimed to verify the agreement between Tracheal wash and BALF cytology in assessing EIPH in racehorses. For this purpose, cytological data from 172 patients regarding hemosiderophage percentage, hemosiderin score, and percentage of recent, intermediate, and old EIPH were reviewed, and the simplified Total Hemosiderin Score (sTHS) was calculated. Non-parametric statistical tests were used to assess the difference and the correlation between TW and BALF. The two cytological methods strongly agreed in evaluating EIPH in racehorses for hemosiderophage percentage (ρ = 0.89, *p* < 0.001), hemosiderin score (k = 0.63, *p* < 0.001), sTHS (ρ = 0.87, *p* < 0.001), percentage of recent EIPH (ρ = 0.95, *p* < 0.001), intermediate EIPH (ρ = 0.92, *p* < 0.001), and old EIPH (ρ = 0.85, *p* < 0.001). In conclusion, TW showed to be a reliable method, which might substitute BALF in assessing EIPH in racehorses.

## Simple Summary

Bronchoalveolar lavage fluid is considered the most sensitive technique to diagnose Exercise-Induced Pulmonary Hemorrhage (EIPH) in horses; however, it is sometimes perceived as less practical and more invasive compared to tracheal wash collection. The present retrospective study aimed to assess the agreement between tracheal wash and bronchoalveolar lavage fluid cytology in the evaluation of EIPH in 172 poorly performing racehorses. The two cytological methods showed a strong correlation in assessing hemosiderophage percentage, hemosiderin score, simplified Total Hemosiderin Score, and percentage of recent, intermediate, and old EIPH. In conclusion, tracheal wash cytology was found to be a reliable tool, which might be used in place of bronchoalveolar lavage fluid cytology for the assessment of EIPH in racehorses.

## 1. Introduction

Exercise-induced pulmonary hemorrhage (EIPH) is a common pulmonary disease defined as bleeding occurring within the lungs during strenuous exercise and is a highly frequent condition among racehorses (Standardbreds and Thoroughbreds) [1]. The diagnosis of EIPH is based on the detection of blood in the tracheal lumen within 120 min after strenuous exercise and/or the presence of hemosiderophages in the bronchoalveolar lavage fluid (BALF) [2,3]. Using tracheobronchoscopy, the prevalence of EIPH in racehorses ranges between 44% and 75%, whereas it ranges between 90% and 100% when the diagnosis is made by BALF cytology [2]. Since the latter allows the identification of occult EIPH and small amounts of blood that might be missed using tracheobronchoscopy, it is considered the most sensitive method to diagnose EIPH [2,4]. However, a survey submitted to veterinary clinicians in the UK raised some concerns among trainers about BALF collection in racehorses in training activity. This procedure is perceived as less practical and more invasive compared to the tracheal wash (TW) collection [5,6]. In addition, it is often less accepted by trainers because it requires sedation and, consequently, an interruption of the horse training for some days afterward [5]. The same research reported that even many veterinary clinicians themselves, especially in the UK, consider the TW more appropriate for time-efficient health screening [5]. In the literature, several studies have compared TW and BALF cytology to identify the agreement between the two methods for respiratory disease diagnosis. TW was considered more sensitive and specific in detecting lower respiratory tract diseases in 154 hospitalized horses with and without respiratory diseases [7]. Other studies compared TW and BALF cytology of racehorses with poor performance, finding a significantly higher percentage of mast cells in BALF, a poor agreement for neutrophils, and no differences in eosinophils [8,9]. When the comparison between TW and BALF was considered in terms of hemosiderophages for the diagnosis of EIPH, no concordance between the two methods was found [10]. However, the latter study has not considered the Total Hemosiderin Score (THS), which is a valid index used to distinguish between EIPH-negative and EIPH-positive horses on BALF cytology [11].

The present study aimed to investigate the agreement between TW and BALF in the evaluation of EIPH in racehorses, particularly in assessing hemosiderophage percentage, hemosiderin score, and Total Hemosiderin Score. Furthermore, since EIPH can be also classified as recent, intermediate, and old based on the color changes of hemosiderin contained in macrophages [12,13], the concordance between TW and BALF cytology in the classification of EIPH as recent (<14 days), intermediate (between 15 and 30 days), and old (>30 days) was verified. Finally, the correlation between age and THS, tracheal blood score (TBS), and THS, and between the days from the last race to hospital admission and the diagnosis of recent, intermediate, or old EIPH on both TW and BALF was investigated.

## 2. Materials and Methods

### 2.1. Animals

The animals included in this study were retrospectively selected among client-owned horses referred to the Equine Veterinary Hospital of the University of Milan (Italy) for poor performance evaluation, between January 2001 and December 2023. Inclusion criteria were the presence of TW and BALF cytology collected in the same instance in racehorses (Standardbred and Thoroughbred). As part of the hospital’s routine admission, an informed consent was signed by the owner for each horse. Being a retrospective study, all data were processed following the current international legislation regarding ethical research on animals, without requiring a submission of the protocol to the Institutional Animal Care and Use Committee.

### 2.2. Diagnostic Protocol

Horses admitted for poor performance evaluation underwent a diagnostic protocol including resting examination, standardized incremental treadmill test for the evaluation of lactate curve, high-speed treadmill endoscopy, post-exercise tracheobronchoscopy (30 min after the end of high-speed treadmill test), bronchoalveolar lavage (BAL), and tracheal wash (TW) [14,15,16]. The presence of blood in the trachea and the mainstem bronchi was graded from 0 to 4 based on the scoring system already reported in the literature (tracheal blood score, TBS) [4]. TW and BALF were collected as previously described [14,15]. Briefly, the horses were restrained into the stocks, sedated with detomidine hydrochloride (0.01 mg/kg IV), and a nose twitch was applied. A 13 mm diameter 170 cm long flexible video endoscope (EC-530WL-P, Fujifilm, Tokyo, Japan) was passed through one of the nostrils, into the pharynx, and then inserted into the trachea. To collect BALF, a sterile catheter was put into the service canal of the endoscope and 60 mL of a 0.5% lidocaine hydrochloride solution was sprayed at the level of the tracheal bifurcation to reduce the coughing reflex. The endoscope was then passed through the right main bronchus until it was wedged and here, a total of 300 mL of sterile 0.9% saline solution was instilled using the sterile catheter. The fluid was immediately re-aspirated. To collect TW, 60 mL of sterile 0.9% saline solution was flushed into the intrathoracic portion of the trachea and re-aspirated. Samples were stored in sterile ethylenediaminetetraacetic acid (EDTA) tubes for cytology. A few drops of BALF and TW samples were cytocentrifuged (Rotofix 32, Hettich Cyto System, Tuttlingen, Germany) for 5 min at 500 rpm. The slides were air dried, stained with May-Grünwald Giemsa (MGG) and Perl’s Prussian Blue (PPB), and then observed under a light microscope at 400× and 1000× for 400 cell differential counting [17]. The percentage of hemosiderophages of the total macrophages was calculated and the hemosiderin was scored from 0 to 4 based on the blue coloration of the macrophages’ cytoplasm [18]. Based on the color changes of hemosiderin contained in macrophages, hemosiderophages reflect the presence of recent (free or phagocytosed red blood cells and/or hemosiderin as finely granular yellowish-light brown pigments; onset time < 14 days), intermediate (more compact refractive green hemosiderin; onset time between 15 and 30 days), and/or old (dark green-brown hemosiderin, very compact and irregularly spherical; onset time > 30 days) episodes of EIPH. Each horse may be affected by a single episode (recent, intermediate, or old) or multiple episodes of pulmonary hemorrhage that have occurred over time [12,13]. One hundred macrophages were assessed, and the percentage of hemosiderophages of the total macrophages showing recent, intermediate, and old EIPH was reported.

### 2.3. Retrospective Data Evaluation

The tracheobronchoscopy records were reviewed for the tracheal blood score (TBS). The cytology records (BALF and TW) were reviewed for the percentage of hemosiderophages, the hemosiderin score, and the percentage of recent, intermediate, and old EIPH. For the calculation of the Total Hemosiderin Score, the percentage of hemosiderophages was multiplied by the median hemosiderin score to obtain a simplified Total Hemosiderin Score (sTHS), with a maximum score of 400 [3]. The official racing database UNIRE was reviewed to record the date of the last race before hospital admission [19].

### 2.4. Statistical Analysis

All data were organized in a spreadsheet (Microsoft Excel, Office package) and analyzed using the statistical software IBM SPSS v. 29.0 (IBM Corp., Armonk, NY, USA). The Shapiro–Wilk test was used to evaluate the normality of data distribution. Since data were not normally distributed, they were reported as median (25th percentile–75th percentile). Categorical variables were reported as percentages. To evaluate the difference and the correlation between TW and BALF for hemosiderophage percentage, sTHS, and percentage of recent, intermediate, and old EIPH, the Mann–Whitney U test and the Spearman’s correlation were performed respectively. The Spearman’s rho (ρ) coefficient was interpreted as follows: <0.10 as negligible correlation, 0.10–0.39 as weak correlation, 0.40–0.69 as moderate correlation, 0.70–0.89 as strong correlation, 0.90–1.00 as very strong correlation [20]. To evaluate the agreement between TW and BALF for the hemosiderin score, Cohen’s Kappa test was used. The Cohen’s coefficient (k) was interpreted as follows: ≤0.20 as no agreement, 0.21–0.40 as weak agreement, 0.41–0.60 as moderate agreement, 0.61–0.80 as strong agreement, 0.81–0.99 as very strong agreement, and 1 as perfect agreement.

Spearman’s correlation was used to evaluate the correlation between the days from the last race to hospital admission and the percentage of recent, intermediate, or old EIPH, the correlation between age and sTHS, and the correlation between TBS and sTHS.

#### ROC Curve

The sTHS cutoff value of 75 on BALF cytology was used to classify horses in EIPH-negative (sTHS ≤ 75) and EIPH-positive (sTHS > 75) [11]. Then, this classification was used to build an ROC (receiver operating characteristic) curve to determine a cutoff value for sTHS in TW cytology. The Youden index (J) was used to choose the cutoff point that maximized the sensitivity and specificity. The AUC (area under the curve) was calculated and interpreted using the following criteria: 0.5 non-informative test, 0.5 < AUC ≤ 0.7 less accurate test, 0.7 < AUC ≤ 0.9 moderately accurate test, 0.9 < AUC < 1 highly accurate test, and AUC = 1 perfect test. Test performance criteria were determined with a contingency table to assess the sensitivity and specificity values of sTHS in TW. A *p*-value < 0.05 was considered statistically significant.

## 3. Results

### 3.1. Animals

Among a population of 475 racehorses admitted to the Veterinary Hospital for poor performance evaluation, 172 met the inclusion criteria. The studied population included 20 Thoroughbreds (11.6%) and 152 Standardbreds (88.4%). A total of 63 were females (36.6%), 93 were males (54.1%), and 16 were geldings (9.3%). The median age was 3 years old (3–4 years old), and the median bodyweight was 452 kg (435–479 kg).

### 3.2. Statistical Results

By tracheobronchoscopy, 83/172 horses had a TBS of 0 (48.3%), 36/172 horses had a TBS of 1 (20.9%), 10/172 horses had a TBS of 2 (5.8%), 38/172 horses had a TBS of 3 (22.1%), and 5/172 horses had a TBS of 4 (2.9%). Hemosiderophages were found in 162/172 horses (94.2%) by TW, and in 158/172 horses (91.9%) by BALF cytology.

Out of 162 horses that had hemosiderophages in TW cytology, 17/162 horses (10.5%) had only a recent episode of EIPH, 6/162 horses (3.7%) had only an intermediate episode of EIPH, no horses (0%) had only an old episode of EIPH, while 139/162 horses (85.8%) showed the presence of multiple episodes of EIPH. Out of 158 horses that had hemosiderophages in BALF cytology, 20/158 horses (12.7%) had only a recent episode, 6/158 horses (3.8%) had only an intermediate episode, 1/158 horses (0.6%) had only an old episode of EIPH, while 131 horses (82.9%) showed the presence of multiple episodes of EIPH.

Median, 25th percentile, and 75th percentile for the percentage of hemosiderophages, simplified Total Hemosiderin Score, and percentage of recent, intermediate, and old EIPH are reported in Table 1. The number and percentage of horses for each hemosiderin score are reported in Table 2.

No statistically significant difference (*p* > 0.05) between TW and BALF was found for hemosiderophage percentage, sTHS, and percentage of recent, intermediate, and old EIPH. A strong correlation between TW and BALF was found for hemosiderophage percentage (ρ = 0.89, *p* < 0.001), sTHS (ρ = 0.87, *p* < 0.001), and percentage of old EIPH (ρ = 0.85, *p* < 0.001); and a very strong correlation was found for the percentage of recent EIPH (ρ = 0.95, *p* < 0.001) and intermediate EIPH (ρ = 0.92, *p* < 0.001). A strong agreement was found for the hemosiderin score (k = 0.63, *p* < 0.001) between TW and BALF.

A negligible correlation was found between age and sTHS (ρ = 0.13, *p* = 0.09 for TW; ρ = 0.05, *p* = 0.53 for BALF), as well as between the days from last race to hospital admission and percentage of recent EIPH (ρ = 0.15, *p* = 0.06 for TW; ρ = 0.09, *p* = 0.29 for BALF), intermediate EIPH (ρ = −0.1, *p* = 0.23 for TW; ρ = −0.08, *p* = 0.36 for BALF), or old EIPH (ρ = −0.08, *p* = 0.31 for TW; ρ = −0.02, *p* = 0.85 for BALF). A weak correlation was found between TBS and sTHS (ρ = 0.31, *p* < 0.001 for TW; ρ = 0.35, *p* < 0.001 for BALF).

#### ROC Curve

**Figure 1:**
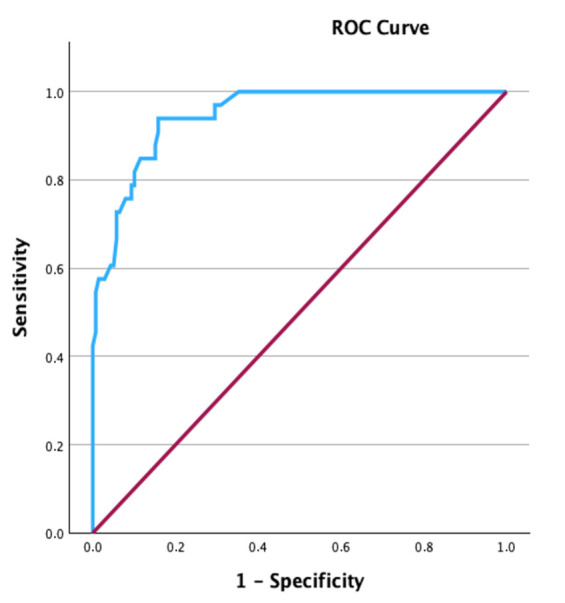
Receiver Operating Characteristic (ROC) curve, showing the sensitivity and the 1 minus specificity (1 − Specificity) for different simplified Total Hemosiderin Score cutoff values in the tracheal wash. The blue line represents the ROC curve, and the red line represents a reference line.

Based on the sTHS cutoff value of 75 on BALF cytology [11], 139/172 patients were included in the control group (EIPH-negative), while 33/172 horses were considered affected (EIPH-positive). In the present study, this classification was used to create an ROC curve to determine a possible cutoff value for sTHS in TW cytology. The ROC curve plotted with an optimal sTHS cutoff value of 61 for TW (J = 0.781) and the test showed an AUC of 0.95 (95% Confidence Intervals: 0.913–0.981), indicating a highly accurate test (Figure 1). By using the sTHS cutoff point of 61 for TW, a contingency table was built (Table 3), and the sTHS method was found to show sensitivity and specificity values of 94% and 84% respectively.

## 4. Discussion

The present study demonstrated an agreement between TW and BALF in assessing EIPH in horses. Particularly, the comparison between TW and BALF revealed a strong correlation when evaluating hemosiderophage percentage, hemosiderin score, sTHS, and percentage of recent, intermediate, and old EIPH.

Previously, a study compared TW and BALF cytology for an evaluation of the hemosiderophage-to-macrophage ratio in racehorses; however, no concordance was found between the two methods [10]. This finding could be probably due to the limited number of horses that were diagnosed with EIPH (33 horses by TW, 21 horses by BALF) compared to our study, where 162 horses by TW and 158 horses by BALF had cytological evidence of EIPH. Such a low positivity for EIPH could be related to the fact that the authors did not use the PPB staining method to identify EIPH cytologically, which is specifically used to detect cellular iron content and for counting hemosiderin-laden macrophages [10].

The strong correlation found between TW and BALF cytology for all the parameters that allow the assessment of EIPH (hemosiderophage percentage, hemosiderin score, sTHS, percentage of recent, intermediate, and old EIPH) has high clinical importance in equine medicine, especially in the context of horse racings. Since BALF might be perceived by trainers as an invasive method that requires sedation compared to TW collection, which is usually performed using only a nose twitch as a physical restraint [2,5], the possibility of using the latter instead of BALF to assess and classify EIPH could help veterinarians to reduce trainers’ concerns, simplifying the diagnostic evaluation of EIPH in racehorses. Moreover, since some clinicians may not have an endoscope, TW can be collected using the transtracheal approach as well, which is a relatively non-invasive procedure that can be easily performed in the field, in sedated or non-sedated horses, depending on their temperament [21,22,23].

The Total Hemosiderin Score (THS) is a widely used and validated cytological criterion to distinguish EIPH-negative and EIPH-positive horses by BALF [3,11]. However, no THS cutoff value for TW has been reported in the literature yet. Previously, a study attempted to determine a THS cutoff value using TW cytology in Thoroughbred racehorses but failed, probably due to the limited number of horses included and the method used to define EIPH-affected horses and controls [6].

In our study, a sTHS cutoff value of 61 was found to provide the best compromise between sensitivity (94%) and specificity (84%), better distinguishing EIPH-negative from EIPH-positive horses using TW cytology. However, it is important to highlight that, for research purposes, other more specific or more sensitive sTHS cutoff values for TW may be chosen to better select cases and controls, depending on the research aims. Since the sTHS cutoff of 61 has a comparable sensitivity to that reported by Doucet and colleagues [11], but a lower specificity, it may be possible that a higher number of horses will be classified as EIPH-positive by TW.

The sTHS cutoff value of 61 obtained in our study for TW was lower compared to the sTHS cutoff value of 75 on BALF cytology reported in the literature [11]. This is likely due to the techniques used to collect TW and BALF [14,16]. TW was collected from the trachea, where fluid coming from both lungs accumulates [8,24], while BALF was collected from the lung [2,25]. A previous study comparing BALF cytology from the right and left lungs of 138 French Trotters in training activity reported that 56% of horses would be incorrectly classified as EIPH-negative based on unilateral sampling of the lung [25]. In addition, since TW contains secretion and cells coming from all areas of the lungs [24], it is considered by some authors to be the method that gives a better representation of the whole lung, especially for detecting focal pulmonary diseases [7,24]. Hence, since EIPH is not a diffuse disease but mainly affects the caudo-dorsal region of the lungs [2,26], it might be subject to misdiagnosis when a singular site of the lungs is sampled [2]. For these reasons, it is likely that in some cases the number of hemosiderophages, and consequently sTHS, might be higher in TW compared to BALF cytology.

No correlation between age and sTHS was found, as previously reported in the literature in which no or trivial correlation was reported [3,6,10]. Since most of the horses assessed in our study were aged between 3 and 4 years old, the lack of correlation could be due to the narrow range of ages included. Moreover, career duration, number of starts, and other confounding factors that may have likely influenced the correlation between age and sTHS were not considered in the present study. However, previous research has demonstrated that age may be considered a non-risk factor for EIPH, even when these confounding factors were taken into account [1,27,28,29].

No correlation was found between the days from the last race to hospital admission and the percentage of recent, intermediate, and old EIPH. This finding is likely due to the lack of available data concerning the training workload performed by the horses between their last race and the hospital admission, making it challenging to know whether and how many times horses experienced EIPH in that period.

Finally, a positive but weak correlation was found between TBS and sTHS for both TW and BALF cytology. In the present study, tracheobronchoscopy for TBS evaluation was performed only once, 30 min after the end of the high-speed treadmill test. However, to increase the specificity of the test, it is reported that it may be better to examine the horse at least three consecutive times after the strenuous exercise [2]. Therefore, failing to detect or underestimate blood volume in the trachea could be attributed to the examination timing and/or to the presence of a minimal amount of blood in the lower airways that did not reach the trachea [2]. Moreover, tracheobronchoscopy provides information about the present state of the airways in terms of severity of bleeding after a high-intensity exercise, whereas TW and BALF cytology provide evidence of both recent and old episodes of EIPH, since hemosiderophages can be found for at least 28 days after the bleeding event by cytology [2,4,12]. Considering that, the absence of correlation could be due to the fact that a high sTHS indicates a greater accumulation of hemosiderin into macrophages over time, whereas a high TBS indicates a greater severity of a single bleeding episode. In agreement with our results, some studies have previously assessed the influence of TBS over THS (on TW or BALF cytology), finding no or a weak correlation between these two variables [3,6].

## 5. Conclusions

The present retrospective study demonstrated that TW and BALF agreed in the evaluation and classification of EIPH in poorly performing Thoroughbred and Standardbred racehorses, showing no differences in assessing hemosiderophage percentage, hemosiderin score, sTHS, and percentage of recent, intermediate, and old EIPH. In addition, a sTHS cutoff value of 61 was found to be the best cutoff point to distinguish between EIPH-negative and EIPH-positive horses using TW cytology.

In conclusion, TW cytology was found to be a reliable diagnostic tool that may substitute BALF when assessing EIPH in horses, especially in the context of horse racing where trainers’ concerns about BALF collection and the requirement of sedation are significant. However, EIPH and mild/moderate equine asthma have a high co-incidence in racehorses, and TW is not considered an appropriate alternative to BALF cytology for the characterization of lower airway inflammation [30].

## Figures and Tables

**Table 1:** Median, 25th and 75th percentiles for the percentage of hemosiderophages, simplified Total Hemosiderin Score, and percentage of recent, intermediate, and old EIPH of all horses (n = 172).

	TW	BALF
Hemosiderophages (%)	16 ^1^ (7.3–25.0)	16 ^1^ (6.0–24.5)
sTHS	36 ^2^ (15.3–75.0)	32 ^2^ (12.0–66.0)
Recent EIPH (%)	30 ^3^ (5.0–65.0)	35 ^3^ (10.0–66.3)
Intermediate EIPH (%)	40 ^4^ (10.0–60.0)	40 ^4^ (10.0–60.0)
Old EIPH (%)	9.5 ^5^ (0–21.0)	0.0 ^5^ (0–15.0)

The same superscripts indicate a lack of statistically significant differences (*p* > 0.05) between TW and BALF cytology. BALF, bronchoalveolar lavage fluid; EIPH, exercise-induced pulmonary hemorrhage; TW, tracheal wash.

**Table 2:** Number (%) of horses for each hemosiderin score.

	Hemosiderin Score				
	0	1	2	3	4
TW	10 (5.8%)	6 (3.5%)	68 (39.5%)	69 (40.1%)	19 (11.1%)
BALF	14 (8.1%)	10 (5.8%)	86 (50.0%)	56 (32.6%)	6 (3.5%)

BALF, bronchoalveolar lavage fluid; TW, tracheal wash.

**Table 3:** Contingency table showing test performance criteria for simplified Total Hemosiderin Score, with a cutoff score of 61 for tracheal wash (sensitivity = 94%; specificity = 84%).

	Controls (sTHS ≤ 75 on BALF)	EIPH (sTHS > 75 on BALF)	Total
TW EIPH-negative (sTHS ≤ 61)	117	2	119
TW EIPH-positive (sTHS > 61)	22	31	53
Total	139	33	172

BALF, bronchoalveolar lavage fluid; EIPH, exercise-induced pulmonary hemorrhage; sTHS, simplified Total Hemosiderin Score; TW, tracheal wash.

## Data Availability

Data presented in this study are available on request from the corresponding author.
